# Cryo-EM structure and polymorphism of Aβ amyloid fibrils purified from Alzheimer’s brain tissue

**DOI:** 10.1038/s41467-019-12683-8

**Published:** 2019-10-29

**Authors:** Marius Kollmer, William Close, Leonie Funk, Jay Rasmussen, Aref Bsoul, Angelika Schierhorn, Matthias Schmidt, Christina J. Sigurdson, Mathias Jucker, Marcus Fändrich

**Affiliations:** 1 Institute of Protein Biochemistry, Ulm University, Helmholtzstrasse 8/1, 89081 Ulm, Germany; 2 Department of Cellular Neurology, Hertie Institute for Clinical Brain Research, University of Tübingen, 72076 Tübingen, Germany; 3 German Center for Neurodegenerative Diseases, 72076 Tübingen, Germany; 4 Queensland Brain Institute, The University of Queensland, St Lucia Campus, Brisbane, QLD 4072 Australia; 5 Institute of Biochemistry and Biotechnology, Martin-Luther-University, 06120 Halle (Saale), Germany; 6 Department of Pathology, University of California, San Diego, 9500 Gilman Drive, La Jolla, CA 92093-0612 USA

**Keywords:** Biochemistry, Cryoelectron microscopy, Alzheimer's disease

## Abstract

The formation of Aβ amyloid fibrils is a neuropathological hallmark of Alzheimer’s disease and cerebral amyloid angiopathy. However, the structure of Aβ amyloid fibrils from brain tissue is poorly understood. Here we report the purification of Aβ amyloid fibrils from meningeal Alzheimer’s brain tissue and their structural analysis with cryo-electron microscopy. We show that these fibrils are polymorphic but consist of similarly structured protofilaments. Brain derived Aβ amyloid fibrils are right-hand twisted and their peptide fold differs sharply from previously analyzed Aβ fibrils that were formed in vitro. These data underscore the importance to use patient-derived amyloid fibrils when investigating the structural basis of the disease.

## Abstract (web summary)

Alzheimer’s disease is characterised by the deposition of Aβ amyloid fibrils and tau protein neurofibrillary tangles. Here the authors use cryo-EM to structurally characterise brain derived Aβ amyloid fibrils and find that they are polymorphic and right-hand twisted, which differs from in vitro generated Aβ fibrils.

## Introduction

Alzheimer’s disease (AD) is a progressive neurodegenerative disease that is characterized by the deposition of Aβ amyloid fibrils as well as of neurofibrillary tangles derived from tau protein^1^. Several observations demonstrate that Aβ peptide is crucial for triggering disease onset^2^. Aberrant multiplications of the gene for the Aβ precursor protein (APP) as well as mutations in the APP gene or in genes of enzymes involved in APP processing lead to AD^3^. The peptide and its associated fibrils form parenchymal plaques and vascular amyloid deposits underlying cerebral amyloid angiopathy (CAA)^4^. Aβ deposits can contain different primary structural variants of the peptide. Particularly well investigated are the 40- and 42-residue peptide variants Aβ(1–40) and Aβ(1–42)^5–7^. While Aβ(1–42) peptide is thought to be pathogenic by the formation of toxic fibrillation intermediates^7^, Aβ(1–40) forms fibril deposits that damage the cerebral vessel walls^4^.

Much of the current knowledge on Aβ peptide originates from the analysis of Aβ fibrils and other aggregates that were formed from chemically synthetic or recombinantly expressed Aβ peptide in vitro. These test tube-derived Aβ aggregates are typically rich in β-sheets. Fibrils possess a cross-β structure and Congo red-affinity, similar to amyloid fibrils purified from patient tissue^8–10^. In vitro formed Aβ fibrils are highly polymorphic and a range of different peptide conformations has been found in different fibril morphologies that were formed from different peptide variants or under different conditions of fibril formation^11–19^. So far, however, it is unknown which of these structures, or whether any of these structures, correctly reflects the pathogenic structure of Aβ peptide inside the brain.

To shed some light on this issue we have purified and analyzed the structural features of Aβ amyloid fibrils from the vasculature (meninges) of AD brain. Using cryo-electron microscopy (cryo-EM) we show that Aβ fibrils from brain tissue are polymorphic but share conserved structural features in terms of peptide fold and protofilament (PF) assembly. Most importantly, the observed structure differs profoundly from the known structures of Aβ fibrils that were formed in vitro.

## Results

### Isolation of Aβ amyloid fibrils from AD brain tissue

Aβ amyloid fibrils were isolated from the meninges of three AD patients, termed here AD1 to AD3 (Supplementary Fig. 1a). The patients died at an age of 70−84 years and exhibited Braak stages VI-VIB. All three patients showed similar histopathologic lesions with numerous neuritic plaques and neurofibrillary tangles in the cerebral cortex, and severe amyloid angiopathy in the leptomeningeal and superficial cortical vessels. The gentle purification procedure avoids harsh chemical or physical conditions and was previously described to maintain the linear architecture of the extracted fibrils^20,21^. Transmission electron microscopy (TEM) analysis of negatively stained specimens reveals the elongated morphology of the fibrils in our samples (Supplementary Fig. 1a). Denaturing gel electrophoresis and Coomassie staining show the purity of the fibril protein that migrates at an apparent molecular mass of 4 kDa (Supplementary Fig. 1b). Western blotting with 6E10 anti-Aβ antibody confirms the presence of Aβ (Supplementary Fig. 1c). Mass spectrometry yields intense m/z peaks corresponding to Aβ(1–40), Aβ(1–38), Aβ(2–40), Aβ(1–37), Aβ(1–36), and Aβ(1–39) (Supplementary Table 1, Supplementary Fig. 2), suggesting that chain length variations rather than posttranslational modifications of the amino acid side chains are the most abundant modifications. The Aβ(1–42) levels in these fibril extracts are low (Supplementary Fig. 2), consistent with literature data on meningeal samples^4^. The pattern of Aβ modification is similar for all three patients studied (Supplementary Fig. 2), corresponding to their similar neuropathology.

### Brain-derived Aβ amyloid fibrils are right-hand twisted

**Fig. 1:**
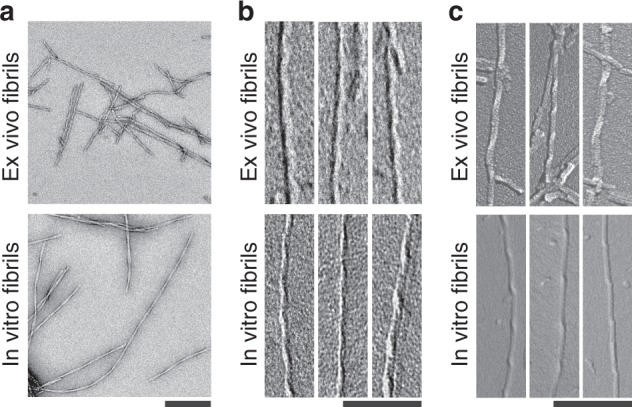
Different structure of brain-derived and in vitro formed Aβ fibrils. **a** Negative stain TEM images of brain-derived amyloid fibrils or in vitro formed Aβ fibrils. Scale bar: 200 nm. **b**, **c** TEM (**b**) and SEM (**c**) images of brain-derived amyloid fibrils or in vitro formed Aβ fibrils after platinum side shadowing. Scale bars: 100 nm

Brain-derived fibrils possess a twisted morphology with well-resolved cross-overs, reminiscent of in vitro formed Aβ fibrils (Fig. 1a). The handedness of their twist is right-handed as demonstrated by platinum side shadowing and analysis of the specimens with TEM or scanning electron microscopy (SEM) (Fig. 1b, c). A right-hand twist was observed consistently with the fibril extracts from all three AD patients analyzed in this study (Supplementary Fig. 3) and differs sharply from the left-hand twist of in vitro formed Aβ fibrils (Fig. 1b, c). Aβ fibrils from brain are also proteinase K resistant and survive proteolytic conditions that readily degrade in vitro formed Aβ fibrils (Supplementary Fig. 4).

### Cryo-EM structure of fibril morphology I

**Fig. 2:**
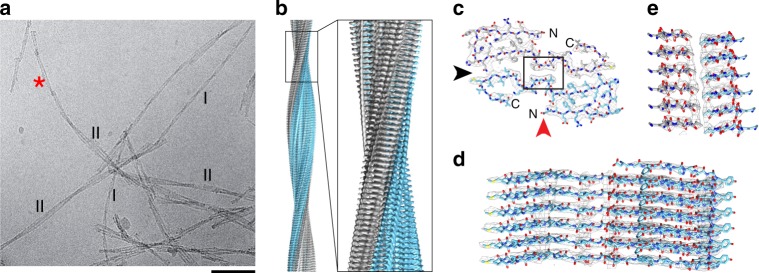
Cryo-EM structure of fibril morphology I. **a** Representative cryo-EM micrograph indicating fibril morphologies I and II. Asterisk: morphology I-like fibril emanating from morphology II. Scale bar: 50 nm. **b** Side view of the reconstructed 3D map. **c** Cross-sectional view of one molecular layer of the fibril superimposed with the molecular model. **d** Side view of the 3D map of a six-layer peptide stack superimposed with the molecular model. The stack is viewed along the red arrow head in panel (**c**). **e** Side view of six molecular layers of the boxed region from panel (**c**), viewed along the black arrow head. The figure illustrates the staggering of the two peptide stacks. The two peptide stacks are colored gray and blue in panels (**b**–**d**)

Cryo-EM shows that brain-derived Aβ fibrils are polymorphic (Fig. 2a). One fibril morphology, termed hereafter morphology I, was relatively abundant in our samples and associated with a relatively small width of 7.4 ± 0.4 nm and a short cross-over distance of 41.5 ± 2.3 nm. The 2D class averages show a staggering of the β-strands in the direction of the fibril *z*-axis (Supplementary Fig. 5a), indicating a pseudo 2_1_-screw axis. Implementing this symmetry, we reconstructed the three-dimensional (3D) map of the fibril at 4.4 Å resolution (Supplementary Table 2) based on the 0.143 Fourier shell correlation (FSC) criterion (Supplementary Fig. 5b, c). The 3D map shows a clear separation of the peptide molecules in the direction of the fibril *z*-axis (Fig. 2b) and allowed us to obtain a valid molecular model of the fibril structure. This model presents a Molprobity score of 2.64, and no C-beta deviations, Ramachandran or rotamer outliers (Supplementary Table 3). 2D projections of the model density are in excellent agreement with the averaged 2D classes and their power spectra (Supplementary Fig. 6). The reconstructed 3D map compared with the model shows a resolution of 4.5 Å (Supplementary Table 3).

**Fig. 3:**
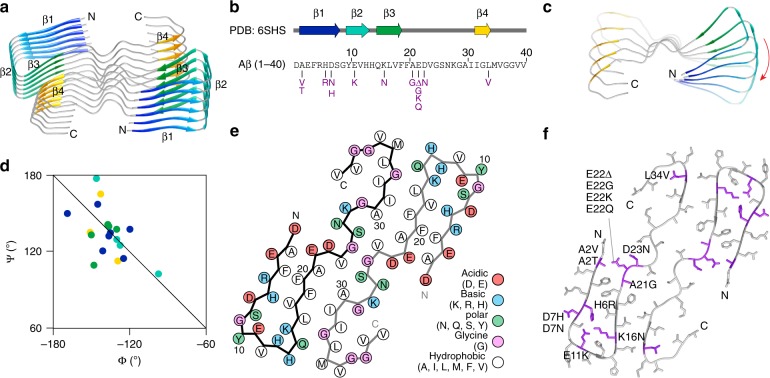
Peptide fold of fibril morphology I. **a** Ribbon diagram of six layers of the fibril. **b** Location of β-strands β1–β4 within the Aβ(1−40) sequence. Mutations are shown in magenta. **c** Top view of a peptide stack showing the right-hand β-sheet twist. Only every sixth molecule shown. **d** Section of the Ramachandran plot showing the Φ/Ψ-pairs of the residues within β-strands β1−β4. The β-strand color coding is kept consistent in panels (**a**–**d**). **e** Packing scheme of one molecular layer of the fibril. **f** Cross-sectional view of the fibril with the known mutational variants highlighted in magenta (3)

The fibril consists of two stacks of peptide. The cross-sectional layers of the fibril contain two Aβ(1–40) molecules that fill the entire density (Fig. 2c, d), demonstrating the absence of major disordered regions. The 3D map is less well-resolved at the peptide C-terminus, consistent with a double-Gly motif at residues 37 and 38 and the C-terminal heterogeneity of the peptide observed by mass spectrometry (Supplementary Fig. 2). Each peptide stack contains four cross-β sheets, termed here β1–β4, that extend between residues 2–8, 10–13, 15–19 and 32–34 (Fig. 3a, b). The β-sheets are uniformly parallel with respect to the orientation of the hydrogen-bonded β-strands relative to one another (Fig. 3a). The β-sheet twist is right-handed (Fig. 3c), whereby the direction of the twist is defined by looking along the main fibril axis. This property of brain-derived Aβ fibrils differs sharply from in vitro formed Aβ(1–40) or Aβ(1–42) fibrils that possess left-hand twisted β-sheets^8,14,19,22^ and from the canonical orientation of the β-sheet twist in globular proteins is also left-hand according to the above definition^23^.

The backbone Ψ/Φ dihedral angles of the β-strands show a distribution within the Ramachandran plot that extends strongly to the left side of the diagonal (Fig. 3d). Such a distribution is characteristic for a right-hand twisted β-sheet^23^. The average of the Ψ + Φ sum of the residues within the four β-sheets is negative (−2.2 ± 17.9°) and thus similar to the value of −2 ± 20° obtained with the right-hand twisted amyloid fibril from human systemic AA amyloidosis^24^. By contrast, the left-hand twisted amyloid fibril from murine systemic AA amyloidosis shows a positive value of +8 ± 20°^24^. The correspondence of the model’s backbone conformation with the right-hand twist of the fibril (Fig. 1b, c, Supplementary Fig. 3) lends further credibility to the correctness of the model.

The peptide fold is C-shaped. Its N- and C-terminal ends form arches, in which the peptide chain is folded back onto the central peptide domain. The latter is buried in the fibril core and forms the interaction sites between the two peptide stacks (Fig. 3e). The contact sites contain hydrophobic and polar amino acid residues (Fig. 3e). The central contact site, which occurs close to the fibril main axis, is formed by residues 24–26 (Fig. 3e). The two equal contact sites at outer radial position involve cross-stack heterotypic zippers between sheets β3 and β4 (Fig. 3a). The contact sites are separated by two small cavities that occur at the interface between the two peptide stacks. The stacks are offset from one another by ~2.41 Å (Fig. 2e), corresponding to the fibril pseudo 2_1_-screw symmetry. Most of the known mutational variants of Aβ peptide^3^ affect the N-terminal arch of the peptide (Fig. 3f).

The N- and C-terminal ends of the peptide are more solvent exposed and accessible than the central domain (Fig. 3e), which is consistent with the proteolytic modification of terminal ends demonstrated by mass spectrometry (Supplementary Fig. 2). The majority of the charged amino acid side chains are solvent exposed, except from a cluster containing His6, His13, Glu11 and Lys16 that is buried within the N-terminal arch. Glu11 and Lys16 are oriented such that they can form salt bridges with one another. Each Aβ molecule shows a ~6 Å height change in the direction of the fibril *z*-axis (Supplementary Fig. 7a) that sterically interdigitates the fibril and produces different tip structures at either fibril end. The height change is specifically prominent within the N-terminal arch where it leads to intermolecular interactions between strand β1 from layer *i* with strand β3 from the layer *i* + 1 (Supplementary Fig. 7b).

### Polymorphism of brain-derived Aβ fibrils

**Fig. 4:**
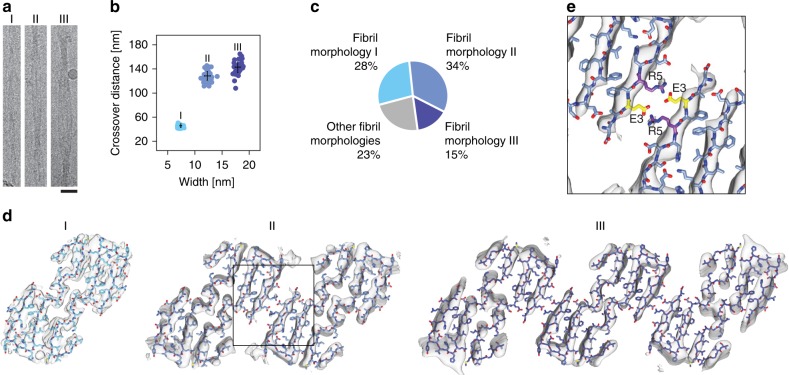
Polymorphism of brain-derived Aβ fibrils. **a** 300 kV Cryo-EM images of fibril morphologies I−III. Scale bar: 20 nm. **b** Crossover distance and fibril width values of fibril morphologies I−III (*n* = 30). Black cross: average value and standard deviation. **c** Relative abundance of fibril morphologies I−III in our sample. **d** Cross-sectional densities of fibril morphologies I−III (gray) superimposed with the molecular model obtained with fibril morphology I. **e** Close-up of the PF−PF interface of fibril morphology II, indicated by a box in panel (**d**), showing the juxtaposed residues Glu3 and Arg5

Besides morphology I, we could discern two other abundant fibril morphologies of our samples, termed here morphologies II and III (Fig. 4a). Morphology II shows a width of 12.3 ± 0.6 nm and a cross-over distance of 129.0 ± 10.5 nm, while morphology III possesses a width of 17.9 ± 0.6 nm and a cross-over distance of 142.8 ± 12.3 nm (Fig. 4b). The three fibril morphologies account for more than 75% of the fibril structures observed in our samples (Fig. 4c) and are present in the fibril extracts of all three AD patients investigated in this study (Supplementary Fig. 8). Cryo-EM reconstructions of the 3D maps of morphologies II and III (Supplementary Fig. 5) achieved spatial resolutions of 5.65 and 7.01 Å (Supplementary Table 2). A pseudo-2_1_ screw symmetry was implemented in these reconstructions as suggested by the staggering of the strands in the 2D class averages (Supplementary Fig. 5a). The two fibril morphologies are polar and contain similarly shaped PFs. Each PF has a cross-sectional dimension of 4.1 × 7.6 nm, which corresponds to the dimensions of the entire cross-section of morphology I (Fig. 4d). The internal structure of the PFs of morphologies II and III is similar to the cross-sectional details seen in the 3D map of fibril morphology I (Supplementary Fig. 5b).

The peptide model of fibril morphology I produces an excellent fit to the 3D maps of fibril morphologies II and III (Fig. 4d). Although the resolution of our data do not suffice to reveal any small structural rearrangements of the peptide conformation in response to their different structural environments, it is evident that the three fibril morphologies can be explained based on the same fundamental Aβ peptide conformation. Therefore, the polymorphism of brain-derived Aβ amyloid fibrils arises primarily from variations in the number of similarly structured PFs. Fibril morphology I contains one PF, while fibril morphologies II and III contain two or three PFs, respectively. The conclusion that the PF structure or peptide fold is conserved in different fibril morphologies is further supported by evidence that filaments corresponding to morphology I can emanate from the terminal ends of morphology II (Fig. 2a). Fibril morphologies II and III possess very similar PF–PF interfaces. These interfaces are formed by a face-to-face packing of the sheets β1 from the adjacent PFs. The β-sheets are oriented such that residues Glu3 and Arg5 from adjacent PFs mutually compensate their charges (Fig. 4e), resembling the salt bridges at the PF–PF interface of the murine AA amyloid fibril^24^.

## Discussion

In this study we have analyzed the structural morphology of Aβ amyloid fibrils from AD brain tissue. We show that brain-derived Aβ fibrils consist of multiple fibril morphologies. In case of fibril morphology I, we obtained a structural model that establishes the fold of the peptide, the hierarchical assembly of the fibril in terms of the presence of two equal peptide stacks, the right-hand fibril twist, the pseudo 2_1_-screw symmetry and the polarity of the fibril. The robustness of the model is confirmed by its correspondence to the experimentally verified 2D class averages and the power spectra (Supplementary Fig. 6), an FSC value of 4.5 when compared to the 3D map (Supplementary Table 2) and a Ψ/Φ angle distribution that faithfully reflects the right-hand twisted topology of the brain-derived fibrils (Fig. 3d). Yet, our model leaves uncertainty regarding the detail geometry of the polypeptide backbone and of the amino acid side chains. The observed C-shaped peptide fold is novel and differs from previously described Aβ structures that were formed in vitro (Supplementary Fig. 9). Unique features of our fibril structure are the N-terminal arch and the central position of residues 24–26 (light green in Supplementary Fig. 9) which contrasts to previously described Aβ fibrils where residues 30–42 form the most central structural elements (yellow-red in Supplementary Fig. 9).

The peptide fold in morphology I fits well to the 3D maps obtained with fibril morphologies II and III. Although the 3D maps of the latter possess lower resolutions than morphology I, it is clear that general PF structure is conserved in all three fibril morphologies and that their main difference is the number of PFs. The three fibril structures account for the majority of fibrils visible in our sample (Fig. 4c), demonstrating that the described structure is representative for Aβ amyloid fibrils from patient brain. The three fibril morphologies were found consistently in the fibril extracts from all three AD patients analyzed in this study. Hence, they are not patient-specific. Yet, it is not possible to exclude that other Aβ fibril morphologies may exist in the brain, for example, in patients affected by certain mutational variants of the peptide.

Fourteen mutations have been described within the sequence of Aβ peptide^3^. Twelve of these mutations are reported to promote AD and/or CAA, while Ala2Thr is protective and His6Arg of uncertain relevance. Projected onto our structure, most mutations have no obviously stabilizing or destabilizing effect (Fig. 3f). This observation is consistent with cell biological studies showing that they may rather affect the proteolytic generation of Aβ peptide. Alternatively, some mutations may simply promote aggregation in general rather than a specific fibril morphology. Only three mutations induce or remove contacts, which could be expected to destabilize the structure: Glu11Lys and Lys16Asn destroy the salt bridge within the N-terminal arch, whereas Leu34Val removes a methylene group within the C-terminal arch. However, the former two mutations were described to act on the proteolytic processing of Aβ^3^, while the latter mutation promotes CAA rather than preventing it.

A particularly interesting structural feature of the observed brain-derived fibrils is their right-hand twist. This property differs strongly from the left-hand twist of previously described Aβ(1–40) and Aβ(1–42) fibrils that were formed in vitro^8–10,12,14,19,22^ and, even more remarkably, from globular protein structures that present a strong preference for oppositely twisted β-sheets^23^. The current study constitutes the second case of a human-pathogenic fibril protein is associated with a right-hand twisted amyloid fibril in vivo. The first one is serum amyloid A protein from human systemic AA amyloidosis (common variant)^24^. Although the molecular origins of the right-hand twist remain to be established, the distribution of the Ψ/Φ-pairs in the Ramachandran plot of Aβ (Fig. 3d) and AA amyloid fibrils^24^ implies that the fold of the fibril protein induces a right-hand twist. Consistent with this notion, our peptide fold differs substantially from the peptide fold observed in the previously described left-hand twisted Aβ fibrils (Supplementary Fig. 9).

These findings underscore the importance of working with patient-derived amyloid fibrils when investigating the structural basis of disease. Similar conclusions were reached previously with several other fibril proteins, which were also found to produce fibril structures in vitro that were morphologically different from their human pathogenic counterparts^21,25^. However, it would be a premature conclusion to state that in vitro formed fibrils are necessarily different from patient fibrils. Instead, we conclude that the observed differences reflect, in these specific cases, the use of fibril formation conditions in vitro that did not exactly match the conditions of fibril formation in vivo. As a consequence, they led to different fibril morphologies, consistent with the idea that the amyloid structure is determined by the host and seed^26^.

Taken together with several recent publications, our data support the concept that pathology arises from specific fibril morphologies or fibril protein conformations. First, while it is clearly established through previous Aβ in vitro fibrillation studies that the peptide is able to adopt a wide spectrum of morphologically different fibril structures^11,13–19^, we show here that pathology is associated with only some, and structurally related, fibril morphologies. These fibril morphologies occur consistently in the fibril extracts from patients that present the same neuropathology (Supplementary Fig. 8). Similar observations have been reported for other fibril systems, including human ATTR and AA amyloidosis, and tau-dependent neuropathology^27–29^. Even mice affected by systemic AA amyloidosis are associated with consistent fibril morphologies in different animals^21^. Second, a recent cryo-EM structure of the murine AA amyloid fibril provided evidence that the resistance of some mouse strains to systemic AA amyloidosis does not arise from a general inability of the variant serum amyloid A proteins to form cross-β fibrils but rather from their incompatibility with the pathologically relevant fibril morphology^24^. Third, different variants of a disease can be associated with different fibril morphologies. Examples hereof are the type A and B amyloid fibrils in hereditary ATTR amyloidosis^30^, the different fibrils in the ‘common’ and the ‘vascular’ variant of systemic AA amyloidosis^31^ or different tau fibril morphologies in different forms of neurodegeneration^29^. The consequence of these observations is that targeting specific fibril morphologies with appropriately selective inhibitors may represent an attractive strategy to interfere with the disease process. The current study now provides a refined structural information on Aβ.

## Methods

### Collection of human AD tissue and controls

All tissue donors were neurologically and psychometrically studied at the Alzheimer Disease Research Center (ADRC) at the University of California, San Diego (UCSD). Three patients, AD1 (female, 70 years, Braak stage VIB), AD2 (male, 76 years, Braak stage VI) and AD3 (female, 84 years, Braak stage VI), showed severe AD and CAA. In all three cases, affected blood vessels were thickened and hyalinized. Upon autopsy, patient brains were collected by the UCSD ADRC Neuropathology Core. The brain weight was 884 g (AD1), 1134 g (AD2) and 988 g (AD3). The brains were sagittally divided at autopsy. The left hemibrain was fixed in 10% (v/v) formalin in water for neuropathological analysis, while the right hemibrain was frozen at −70 °C. Meningeal tissue overlying the cerebral cortex was collected from AD1−3 and from one control case (female, 90 years, Braak stage IIA/B). The control brain was characterized by some thickened leptomeningeal vessels and either no plaques or a low number of amyloid plaques and tangles. The patient presented acute subdural hematoma and mild AD changes characterized by neuritic plaques with dense cores as well as diffuse plaques and neurofibrillary tangles. No amyloid angiopathy was observed in the neocortex or hippocampus. All procedures were reviewed and approved by the UCSD Institutional Review Board. Tissue donors gave informed consent to brain sample collection for research purposes.

### Fibril extraction from AD tissue

Fibril extraction and the further biochemical analysis were conducted under a valid permission from the Ethical Committee of Ulm University. To purify the Aβ amyloid fibrils from the vascular amyloid deposits, 50 mg meningeal tissue were subjected to a slightly modified previously described extraction protocol^20^. In brief, the tissue was homogenized with a scalpel and washed three times with Tris calcium buffer (20 mM Tris, 138 mM NaCl, 2 mM CaCl_2_, 0.1% (w/v) NaN_3_, pH 8.0) followed by centrifugation at 12,000 × *g* for 5 min at 4 °C. The resulting pellet was digested with 5 mg/mL collagenase from *Clostridium histolyticum* (Sigma-Aldrich) overnight at 37 °C in Tris calcium buffer. The sample was centrifuged for 5 min at 12,000 × *g* and 4 °C and the pellet was washed four times with 500 µL ice-cold wash buffer (50 mM Tris, 10 mM ethylendiaminetetraacetic acid, pH 8) followed by centrifugation for 5 min at 12,000 × *g* and 4 °C. The remaining pellet was resuspended in 250 µL ice-cold water and centrifuged for 5 min at 12,000 × *g* and 4 °C. The supernatant (i.e. the fibril extract) was carefully removed and stored at 4 °C. This step was repeated another nine times to generate ten water extracts. This protocol was applied correspondingly to meningeal tissue from the patients AD1−AD3 and meningeal tissues from the control case.

### Formation of amyloid-like fibrils in vitro

Chemically synthetic Aβ(1–40) peptide (Bachem) was incubated in phosphate-buffered saline (PBS, 137 mM NaCl, 2.7 mM KCl, 8 mM Na_2_HPO_4_ and 2 mM KH_2_PO_4_ pH 7.4) for 6 days at 37 °C under constant agitation (100 rpm) with an orbital platform shaker.

### Proteinase K treatment

20 μg/mL brain-derived amyloid fibrils, as quantified by western blot, or 120 μg/mL in vitro formed amyloid-like fibrils were incubated for 30 min at 37 °C with 50 µg/mL proteinase K from *Streptomyces griseus* (Sigma-Aldrich). The digest was constantly agitated at 700 rpm with an orbital platform shaker. After incubation the samples were boiled for 5 min at 95 °C to heat-inactivate the protease.

### TEM analysis of negatively stained samples

For TEM specimen preparation 5 µL of the sample solution were placed onto carbon coated, formvar 200 mesh copper grids (Plano), that were glow discharged with a PELCO easiGlow instrument (TED PELLA). The sample was incubated on the grid for 1 min at room temperature. Excess solvent was soaked away with filter paper (Whatman).The grid was washed three times with 10 µL water and stained three times with 10 µL 2% (w/v) uranyl acetate in water. The dried grids were examined in a JEM-1400 TEM (JEOL) equipped with a F216 camera (TVIPS) that was operated at 120 kV.

### Platinum side shadowing

Formvar and carbon-coated 200 mesh copper grids (Plano) were glow discharged for 20 s at 15 mA using PELCO easiGlow glow discharge cleaning system (TED PELLA). Five microliters of the sample solution was placed onto the grid and incubated for 30 s at room temperature. Excess solution was soaked away using filter paper (Whatman), and the grids were washed three times with 10 µL water and dried at room temperature for 30 min. A 1-nm-thick layer of platinum was evaporated from an angle of 30° onto the grid using a Balzers TKR 010. We examined the grids in a JEM-1400 TEM (JEOL) equipped with a F216 camera (TVIPS) that was operated at 120 kV or by using a Hitachi S-5200 scanning electron microscope (Hitachi) at 10 kV acceleration voltage.

### Cryo-electron microscopy

C-flat holey carbon grids (CF 1.2/1.3-2 C, Electron Microscopy Sciences) were glow-discharged for 40 s at 20 mA using a PELCO easiGlow glow discharge cleaning system (TED PELLA). Four microliters of the fibril extract was applied on the glow discharged grid for 30 s, followed by both side blotting and plunging into liquid ethane. Blotting and plunging was done using a Gatan Cryoplunge 3 (Gatan) operated at 20 °C and >90% relative humidity. To optimize the specimen quality regarding e.g. fibril distribution and ice thickness, the cryo-EM specimens were initially analyzed using a JEM-2100F TEM (Jeol) that was equipped with a DE12 direct electron detector (Direct Electron) and operated at an accelerating voltage of 200 kV. High-resolution data sets for reconstruction of the fibrils were recorded with a Titan Krios (Thermo Fisher Scientific) microscope that was equipped with a K2-Summit detector (Gatan) and operated at an acceleration voltage of 300 kV (see Supplementary Table 2 for further details). The width and crossover distance of the three major morphologies were determined for 30 fibrils each from cryo-TEM images using ImageJ software.

### Reconstruction of the 3D map

The acquired super-resolution movie frames were gain corrected, aligned and binned by a factor of 2 with IMOD^32^. The aligned images were motion corrected and dose weighted using MotionCor2^33^. Gctf was used to estimate the contrast transfer function of the motion-corrected images^34^. The helical reconstruction was performed using RELION 2.1^35^. Fibril segments were manually picked. Segments were extracted with an inter-box distance of ~10% and a box size of ~200, ~240 and ~350 Å for morphologies I, II and III, respectively. Reference-free 2D classification was performed for each morphology separately using a 90% mask in respective of the box size, a regularization value of *T* = 4. Several rounds of 2D class averaging were conducted in that bad classes and class averages which do not belong to the respective morphology were sorted out. 2D class averages showing the helical repeat along the fibril axis were selected and used for the reconstruction. An initial model was generated de novo for all three morphologies from particles selected after 2D class averaging. We refined the particles in a first round of 3D classification with *K* = 1 and *T* = 20. The initial models were low-pass filtered to 60 Å and used as a starting reference. The following initial values were used for twist (morphology I = 2.1°, morphology II = 0.66° and morphology III = 0.61°) and rise (morphology I = 4.84 Å, morphology II = 4.89 Å and morphology III = 4.88 Å). The values were calculated from the crossover distance and the layer line profiles of the 2D classes. The resulting primary models were classified in another round of 3D classification with *K* = 3 (morphology II) or 4 (morphology (I and III) and *T* = 20. We manually selected the class (1 out of 3 or 4) with the clearest β-sheets (*x*−*y* plane) separation and peptide backbone. A pseudo-2_1_ screw axis was assumed for refinement (*K* = 1 and *T* = 20) using initial values of 2.42 Å rise and 181.05° twist for morphology I, 2.445 Å rise and 180.33° twist for morphology II and 2.44 Å rise and 180.305° twist for morphology III. The resulting map was low-pass filtered to 7 Å and used for a final 3D auto-refinement with local optimization of helical twist and rise as well as a T regularization value of 100 for morphology I and *T* = 20 for morphologies II and III. The b-factor for morphology I was automatically calculated by RELION in the post-processing of the reconstructed map. For morphologies II and III b-factor values were manually imposed, because the values obtained by automatic estimation were substantially higher and appeared to cause artefacts.

### Molecular modeling

The sharpened and masked 3D density map of morphology I was used for building a schematic fibril model by using Coot^36^. A poly-alanine model was created de novo along the peptide backbone. The backbone geometries were then refined using Coot and side chains were added to the backbone chain. The orientation of the side-chains was manually refined to avoid atom clashes and Ramachandran outliers in the model. A protein stack consisting of six subunits was assembled and refined with PHENIX^37^ using phenix.real_space_refine^38^ (phenix-1.16-3549). A high-resolution cutoff of 4.4 Å was used to impose noncrystallographic symmetry (NCS) restraints and constraints on all 12 chains. Manually defined cross β-sheet restraints were imposed on the model and further refined using PHENIX. We iteratively corrected steric clashes, Ramachandran and rotamer outliers manually in Coot followed by further refinement using PHENIX. Detailed model evaluation was done using Molprobity^39^ and statistics are shown in Supplementary Table 3.

### Denaturing protein gel electrophoresis

Samples subjected to electrophoresis were prepared by mixing 15 μL of the analyte solution with 5 µL 4× NuPAGE^®^ lithium dodecyl sulfate (LDS) Sample buffer (Life Technologies) and boiling of the mixture for 10 min at 95 °C. Eighteen microliters of the boiled sample was loaded per well of a 4−12% NuPAGE**®** Bis-Tris Gel (17 wells, Life Technologies). As molecular size marker, SeeBlue® Plus2 Pre-Stained Standard (Life Technologies, 6 µL) was used. The gel chamber was filled with NuPAGE**®** MES-SDS Running Buffer (Life Technologies) and the gel was run for 38 min at 180 V.

### Western blot

Proteins separated by denaturing gel electrophoresis were transferred onto a 0.45 µm nitrocellulose membrane (Protran® BA85, Whatman) using a Semi-Dry Blotting System (Biorad). The filter paper (Whatman) was placed into the blotting system and soaked together with the nitrocellulose membrane for 5 min in transfer buffer (20% (v/v) methanol in 1× NuPAGE® Transfer Buffer (Life Technologies)). The blotting chamber was assembled and proteins were blotted at 20 V for 35 min. The membrane was blocked overnight at 4 °C with 50 mL 5% (w/v) milk powder in PBST (PBS + 0.1% (w/v) Tween 20 (Roth)). On the next day, the membrane was incubated for 1 h at room temperature with 1 mL 1:2500 (v/v) murine primary antibody (6E10, Covance) in PBST containing 5% (w/v) milk powder. The membrane was washed thrice with PBST for 5 min each and incubated for 1 h with 1:1000 (v/v) secondary antibody (anti-mouse from goat, horseradish peroxidase conjugated, Dako) in PBST containing 5% (w/v) milk powder. The membrane was washed twice for 5 min in PBST and incubated with SuperSignal West Femto Chemiluminescent Substrate (Pierce) for 5 min. Proteins were visualized by chemiluminescence using a G:Box (Syngene). To quantify Aβ in brain extracts, the staining intensity of the Aβ band in the fibril extract was compared to a standard series containing 1.56 to 100 μg/mL recombinant Aβ(1–40). The band intensity was evaluated with ImageJ software.

### MSD 96-well MULTI-SPOT human (6E10) Aβ triplex assay

The concentrations of Aβ(x-38), Aβ(x-40), Aβ(x-42) in the fibril extracts were determined with the MSD 96-well MULTI-SPOT Human (6E10) Aβ Triplex Assay (Meso Scale Discovery) as described previously^40^. In brief, the fibril extracts were extracted with 70% (v/v) formic acid and sonicated for 35 s on ice. The sample was centrifuged at 25,000 × g for 1 h at 4 °C, and the supernatant equilibrated (1:20) in neutralization buffer (1 M Tris base, 0.5 M Na_2_HPO_4_, 0.05% (w/v) NaN_3_) and diluted up to 1:100 in 1% (w/v) bovine serum album to remain within the quantification range of the assay. Measurements were carried out according to the manufacturer’s instructions using 96-well plates with pre-spotted capture antibodies against Aβ(x-38), Aβ(x-40) and Aβ(x-42). The plates were incubated for 1 h with 1% bovine serum albumin in Tris buffer (w/v) and then washed with Tris buffer. The diluted samples were then added to the plate and incubated with the SULFO-TAG 6E10 antibody solution for 2 h. The wells were washed and MSD Read Buffer T was added immediately prior to analysis with a Sector® Imager 6000 and MSD Discovery Workbench software 2.0.

### Mass spectrometry

An aliquot of the fibril extracts was dissolved in 6 M guanidine hydrochloride, 10 mM Tris buffer, pH 8, to disassemble the fibrils into monomers. The remaining salts were removed using ZipTip columns (Merck Millipore). Matrix-assisted laser desorption/ionization mass spectra were recorded as described previously^41^. Spectra were recorded covering an m/z range from 3000 to 5000.

### Reporting summary

Further information on research design is available in the Nature Research Reporting Summary linked to this article.

## Supplementary information

Supplementary Information

Reporting Summary

Source Data

## Data Availability

The reconstructed cryo-EM maps were deposited in the Electron Microscopy Data Bank with the accession codes EMD-10204 (morphology I), EMD-4864 (morphology II) and EMD-4866 (morphology III). The coordinates of the fitted atomic model were deposited in the Protein Data Bank under the accession code 6SHS (morphology I). The source data underlying Figs. 3d and 4b, c and Supplementary Figs. 1b, c, 2a–c, 4a–c, 5c and 8a–c are provided as a Source Data file. Other data are available from the corresponding author upon reasonable request. The amounts of fibrils that are left over from the three Alzheimer patients, which we used in our study, are limited.
