# Supplementary material for: Cryo-EM structure and polymorphism of Aβ amyloid fibrils purified from Alzheimer’s brain tissue
Source: Nat Commun. 2019 Oct 29;10:4760. doi: 10.1038/s41467-019-12683-8 (PMC6820800; doi:10.1038/s41467-019-12683-8)
Supplement: Supplementary file 1 — Supplementary Information [file 41467_2019_12683_MOESM1_ESM.pdf]

Supplementary Information

Cryo-EM structure and polymorphism of Aβ amyloid fibrils purified from Alzheimer's brain tissue

Marius Kollmer *et al.*

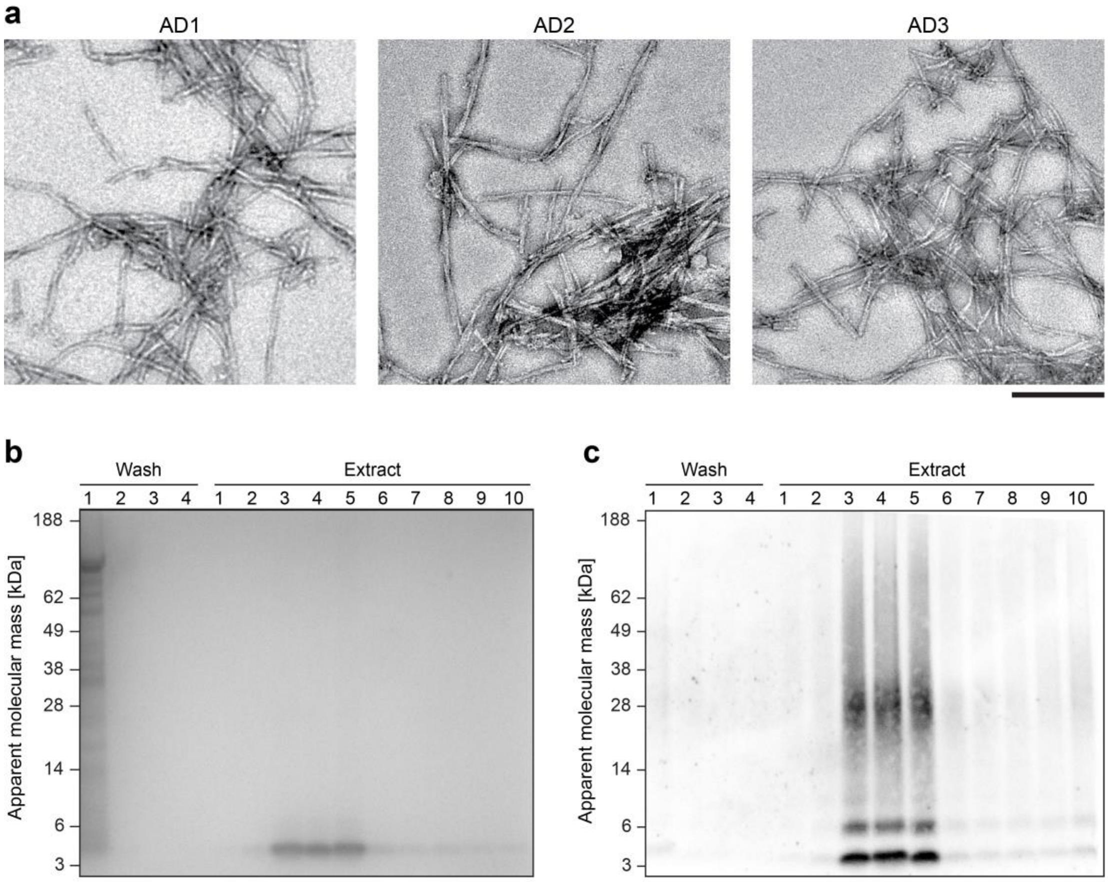

Supplementary Figure 1.

Purification of Aβ amyloid fibrils from AD1, AD2 and AD3.

(a) Negative stain TEM image of the Aβ amyloid fibrils extracted from patients AD1, AD2 and AD3. Scale bar: 200 nm. (b-c) Representative Coomassie-stained denaturing protein gel (b) and western blot (c) of four washing steps with Tris-EDTA buffer and the first ten fibril extraction steps using water. The western blot uses 6E10 primary antibody, which recognizes the Aβ N-terminus.

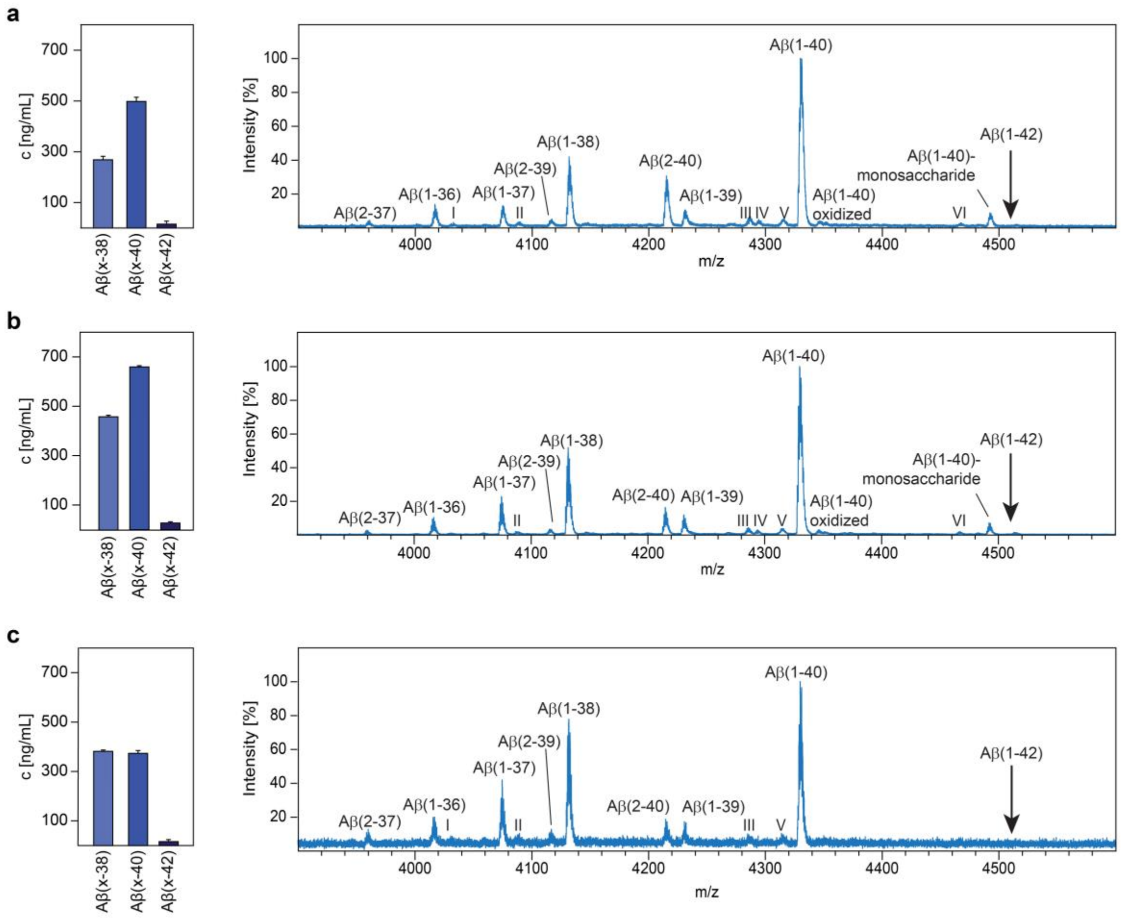

Supplementary Figure 2.

Molecular composition of brain-derived Aβ amyloid fibrils.

(a-c) Constitutional analysis of the fibril extracts from AD1 (a), AD2 (b) and AD3 (c). Left side: the MSD 96-well MULTI-SPOT Human (6E10) Aβ Triplex Assay determines the relative concentrations of Aβ(x-38), Aβ(x-40) and Aβ(x-42) in the fibril extracts. Error bars show standard deviation. Right side: mass spectra of the fibril extracts (see Supplementary Table 1 for details). The expected position of Aβ(1-42) is marked. Roman numbers (I-VI) refer to peaks that could not be assigned (±2 Da mass difference) to any fragment of Aβ(1-43) considering the following possible modifications: pyroglutamylation, nitration, phosphorylation, oxidation and glycosylation.

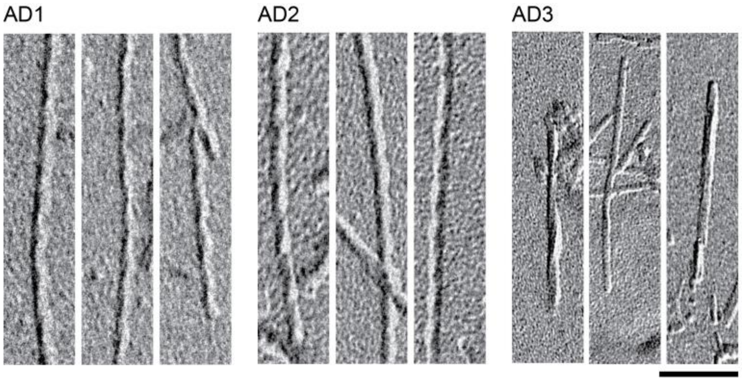

Supplementary Figure 3.

Right hand supertwist of brain-derived Aβ amyloid fibrils from cases AD1-AD3.

TEM images of platinum side shadowed Aβ amyloid fibrils extracted from patients AD1-AD3 as indicated in the figure. Scale bar: 50 nm.

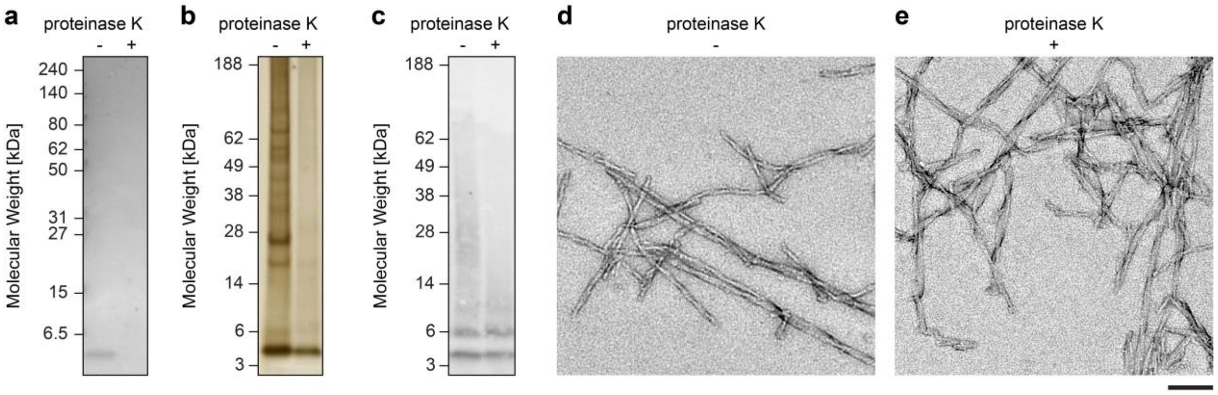

Supplementary Figure 4.

Brain-derived Aβ amyloid fibrils are more proteinase K stable than *in vitro* formed Aβ fibrils.

(a) Coomassie-stained denaturing protein gel of a sample of 120 μg/mL *in vitro* formed Aβ fibrils before and after digestion with 50 μg/mL proteinase K. (b) Silver-stained denaturing protein gel of 20 μg/mL purified AD1 Aβ amyloid fibrils before and after digestion with 50 μg/mL proteinase K. (c) Western blot with 6E10 primary antibody of the same two samples as in panel (b). (d-e) Negative stain TEM images of brain-derived Aβ amyloid fibrils before (d) and after (e) proteinase K digestion. Scale bar: 200 nm.

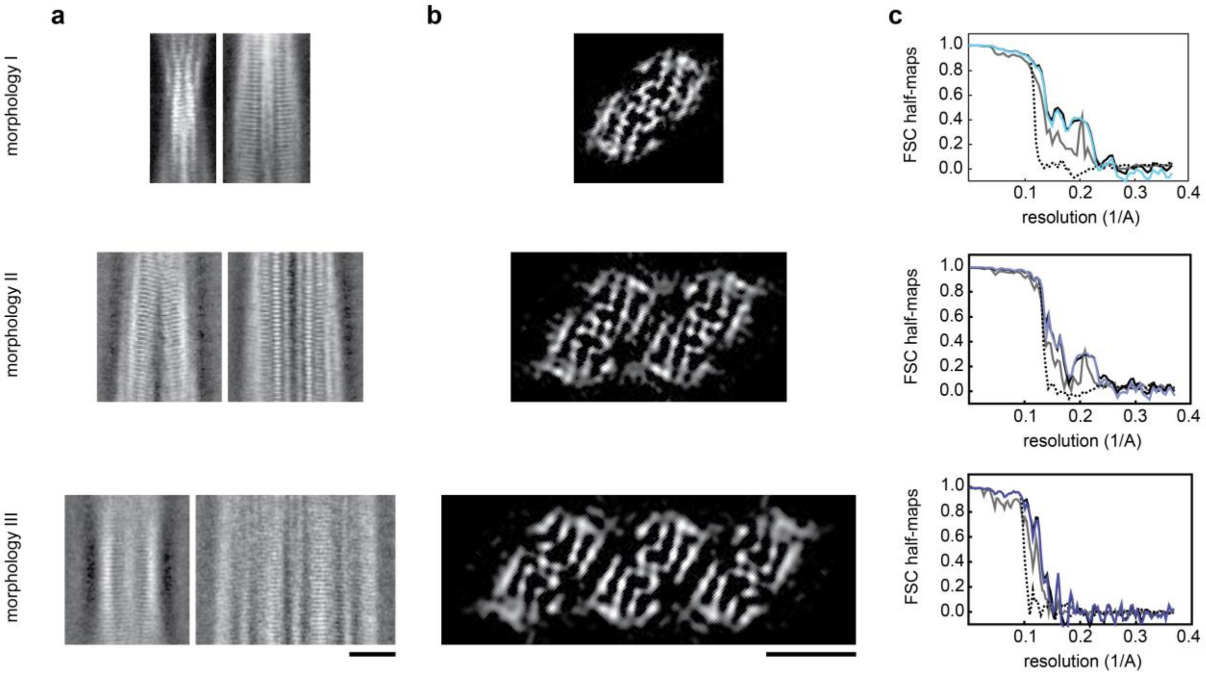

Supplementary Figure 5.

2D class averages and resolution estimates for the 3D density maps of fibril morphologies I-III.

(a) Representative 2D class averages of fibril morphologies I, II and III, taken at different axial positions. The class averages show a staggering of β-strands along the fibril axis and suggest the presence of a pseudo 2_1-screw axis. Scale bar: 5 nm. (b) Cross-sectional density of fibril morphologies I-III. Scale bar: 5 nm (c) FSC between the two half-maps (blue lines = FSC corrected; grey lines = FSC unmasked maps; black lines = FSC masked maps; black dashed lines = Corrected FSC phase randomized masked maps).

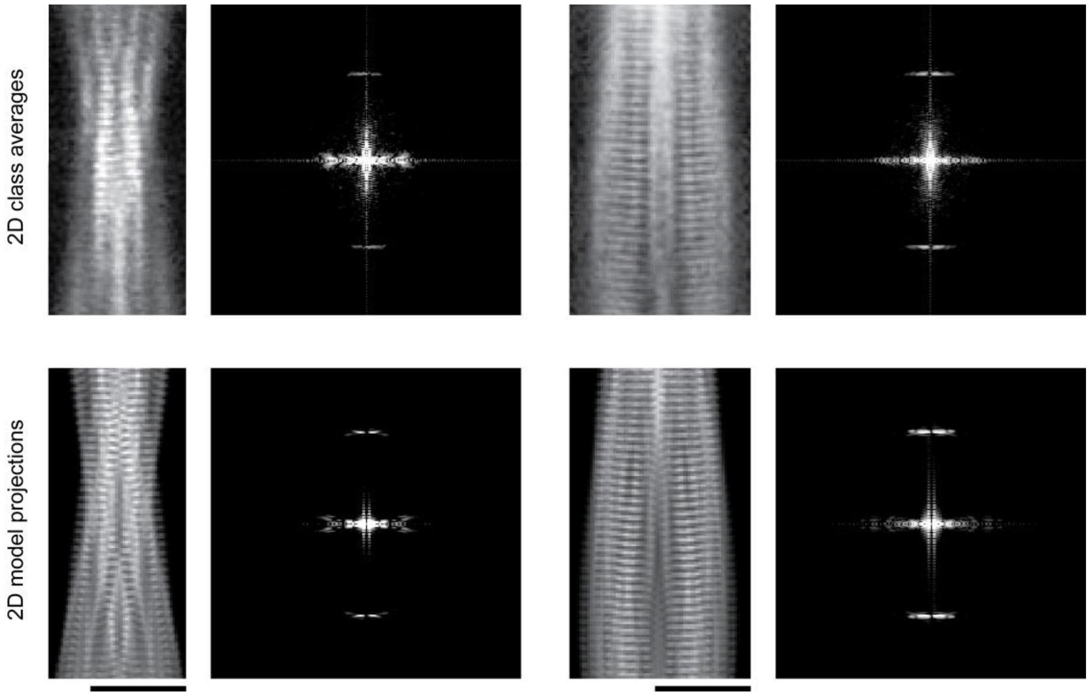

Supplementary Figure 6.

Comparison of the 2D class averages with the model of morphology I.

Side-by-side comparison of 2D class averages and corresponding power spectra (top row) with 2D projections of the model (bottom row). Scale bar: 5 nm.

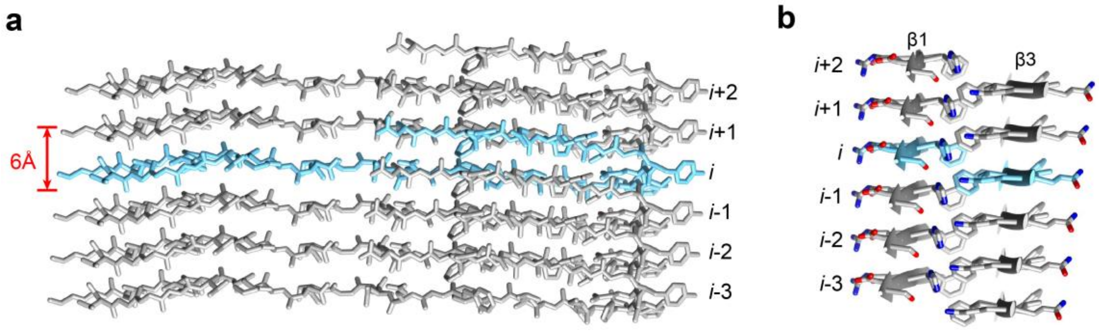

Supplementary Figure 7.

Axial rise of the peptide chain.

(a) Side view of one peptide stack of fibril morphology I. The chain rise per molecule is 6 Å between the carbonyl carbons of Asp1 and His13. (b) Interactions within the N-terminal arch. Strand β1 of layer *i* interacts with strand β3 of layer *i*+1.

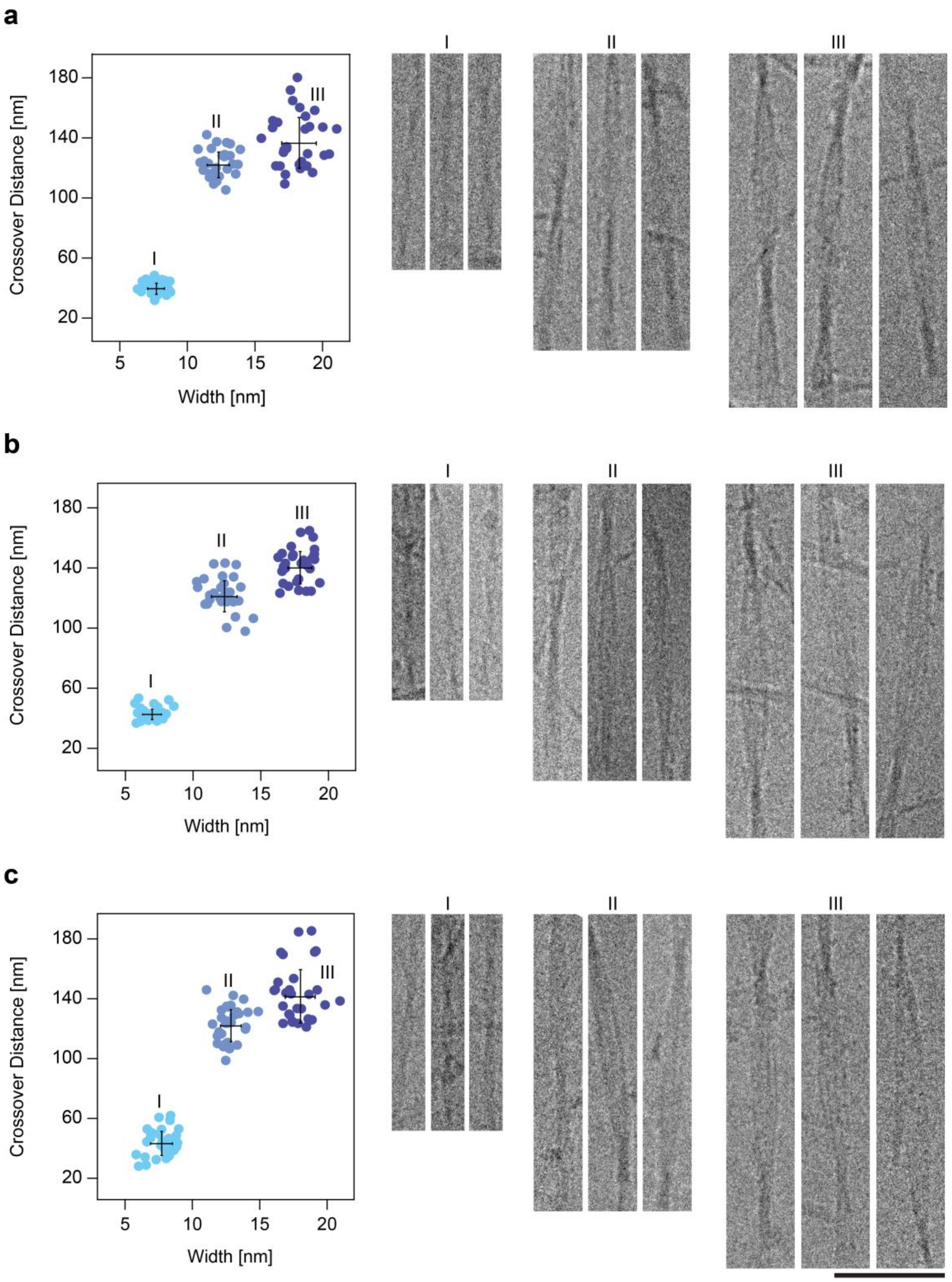

Supplementary Figure 8.

Cryo-EM images of fibril morphologies I-III in cases AD1-AD3.

(a-c) Presence of morphologies I-III from cases AD1 (a), AD2 (b), and AD3 (c). Left side: quantification of the crossover distances and fibril widths based on 200 kV cryo-EM images (n = 30 each). Black cross:

standard deviations and average values. Right side: 200 kV cryo-EM images of morphologies I-III. Scale bar: 50 nm.

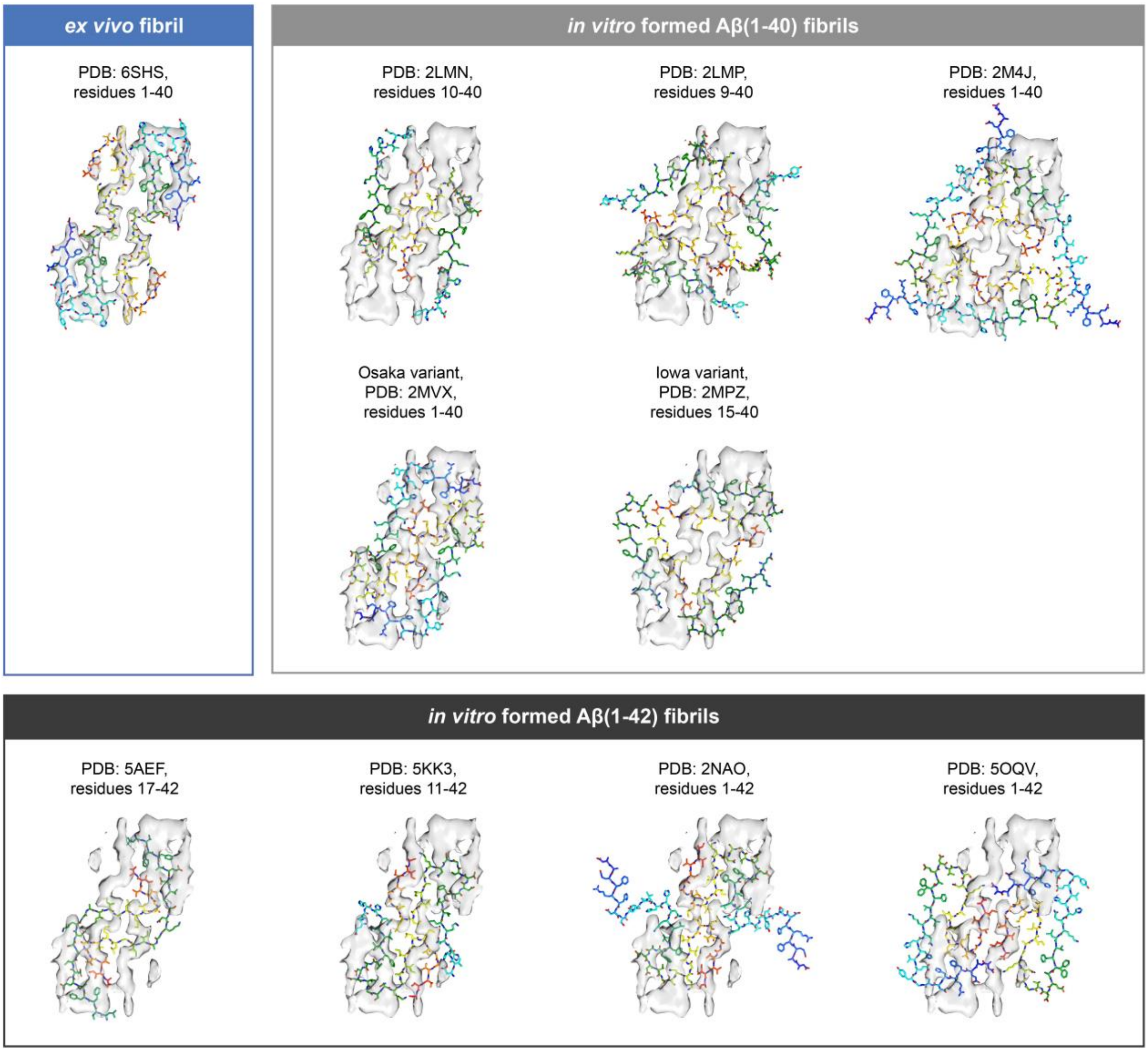

Supplementary Figure 9.

Comparison of our 3D map with structural models of *in vitro* formed Aβ fibrils.

The 3D map of morphology I (grey, in the background) is superimposed with different structural models of *in vitro* formed Aβ(1-40) (light grey box) and Aβ(1-42) fibril (dark grey box)[11,13-19]. Blue box: our structural model. Residue numbers refer to residues in the PDB entry. Two models are based on mutational variants of Aβ (Osaka and Iowa). The Osaka mutation involves the deletion of residue Glu22. Therefore, the PDB entry 2MVX is based on a 39-residue peptide. To avoid confusion in the residue labelling, it is included here within the section Aβ(1-40), as the first and last residues of the peptide correspond to positions 1 and 40 of the wildtype peptide. Side chains in our model are shown in arbitrary conformation.

Supplementary Table 1.

Aβ peptide variants observed by mass spectrometry.

Peak numbers 1-10 and experimental masses refer to the peaks shown in Supplementary Figure 2. Theoretical average masses $[M + H]^{+}$. A possible monosaccharide consistent with peak number 7 is N-Acetyl-D-mannosamine. The theoretical mass given below refers to this modification. Aβ(1-40)oxidized refers to Aβ(1-40), which carries a sulfoxide at position Met35.

Peak number	Experimental mass / Da	Assignment	Theoretical mass / Da
1	4,328	Aβ(1-40)	4,329
2	4,130	Aβ(1-38)	4,131
3	4,213	Aβ(2-40)	4,214
4	4,073	Aβ(1-37)	4,074
5	4,015	Aβ(1-36)	4,017
6	4,228	Aβ(1-39)	4,230
7	4,490	Aβ(1-40)-monosaccharide	4,489
8	3,958	Aβ(2-37)	3,959
9	4,115	Aβ(2-39)	4,115
10	4,343	Aβ(1-40)oxidized	4,347

Supplementary Table 2.

Data collection and reconstruction parameters.

	Morphology 1	Morphology 2	Morphology 3
Microscope	Titan Krios (Thermo Fisher Scientific)		
Camera	K2 Summit (Gatan)		
Acceleration voltage (kV)	300		
Magnification	x 105,000		
Defocus range (µm)	-1 to -3		
Dose rate (e^-/Å/s)	1.7		
Number of movie frames	40		
Exposure time (s)	24		
Total electron dose (e^-/Å)	40.9		
Pixel size (Å)	1.35		
Box size (pixel)	150	180	260
Inter box distance (Å)	20	24	35
Number of extracted segments	80,811	56,410	23,743
Number of segments after 2D classification	48,808	46,630	17,872
Number of segments after 3D classification	12,282	15,345	4,960
Resolution, 0.143 FSC criterion (Å)	4.4	5.65	7.01
Map sharpening B-Factor ($Å^2$)	-145.562	-300	-300
Helical rise (Å)	2.41	2.46	2.45
Helical twist (Å)	181.005	180.339	180.294

Supplementary Table 3.

Modelling parameters.

The provided parameters are based on the fit of morphology I with a six-layer peptide stack, containing 12 peptide molecules.

Non-hydrogen atoms	3660
Number of chains	12
3D map resolution estimates compared with model	4.5
Map CC (around atoms)	0.62
RMSZ bonds (Å)	0.46
RMSZ angles (°)	0.60
All-atom clash score	25.13
Ramachandran outliers/favored (%)	0.00/78.95
Rotamer outliers/favored (%)	0.00/96.77
C-beta deviations (%)	0.00
Molprobity score	2.64
