# Supplementary material for: Cryo-EM structure and polymorphism of Aβ amyloid fibrils purified from Alzheimer’s brain tissue
Source: Nat Commun. 2019 Oct 29;10:4760. doi: 10.1038/s41467-019-12683-8 (PMC6820800; doi:10.1038/s41467-019-12683-8)
Supplement: Supplementary file 2 — Reporting Summary [file 41467_2019_12683_MOESM2_ESM.pdf]

nature research

Corresponding author(s): Marcus Fändrich

Last updated by author(s): Sep 17, 2019

Reporting Summary

Nature Research wishes to improve the reproducibility of the work that we publish. This form provides structure for consistency and transparency in reporting. For further information on Nature Research policies, see Authors & Referees and the Editorial Policy Checklist .

Statistics

For all statistical analyses, confirm that the following items are present in the figure legend, table legend, main text, or Methods section.

n/a	Confirmed	
☐	☒	The exact sample size (*n*) for each experimental group/condition, given as a discrete number and unit of measurement
☐	☒	A statement on whether measurements were taken from distinct samples or whether the same sample was measured repeatedly
☒	☐	The statistical test(s) used AND whether they are one- or two-sided<br>*Only common tests should be described solely by name; describe more complex techniques in the Methods section.*
☒	☐	A description of all covariates tested
☒	☐	A description of any assumptions or corrections, such as tests of normality and adjustment for multiple comparisons
☐	☒	A full description of the statistical parameters including central tendency (e.g. means) or other basic estimates (e.g. regression coefficient) AND variation (e.g. standard deviation) or associated estimates of uncertainty (e.g. confidence intervals)
☒	☐	For null hypothesis testing, the test statistic (e.g. *F*, *t*, *r*) with confidence intervals, effect sizes, degrees of freedom and *P* value noted<br>*Give P values as exact values whenever suitable.*
☒	☐	For Bayesian analysis, information on the choice of priors and Markov chain Monte Carlo settings
☒	☐	For hierarchical and complex designs, identification of the appropriate level for tests and full reporting of outcomes
☒	☐	Estimates of effect sizes (e.g. Cohen's *d*, Pearson's *r*), indicating how they were calculated

Our web collection on statistics for biologists contains articles on many of the points above.

Software and code

Policy information about availability of computer code

Data collection	SerialEM v3.5
Data analysis	RELION v2.1, MotionCor2 v1, Gctf v1.06, IMOD v.4.9.4, COOT v0.8.9, Fiji (ImageJ v.1.51n), Phenix (1.16–3549), Molpropity

For manuscripts utilizing custom algorithms or software that are central to the research but not yet described in published literature, software must be made available to editors/reviewers. We strongly encourage code deposition in a community repository (e.g. GitHub). See the Nature Research guidelines for submitting code & software for further information.

Data

Policy information about availability of data

All manuscripts must include a data availability statement. This statement should provide the following information, where applicable:

- Accession codes, unique identifiers, or web links for publicly available datasets
- A list of figures that have associated raw data
- A description of any restrictions on data availability

The reconstructed cryo-EM maps were deposited in the Electron Microscopy Data Bank with the accession codes EMD-10204 (morphology I), EMD-4864 (morphology II) and EMD-4866 (morphology III). The coordinates of the fitted atomic model were deposited in the Protein Data Bank under the accession code 6SHS (morphology I).

Field-specific reporting

Please select the one below that is the best fit for your research. If you are not sure, read the appropriate sections before making your selection.

Life sciences study design

All studies must disclose on these points even when the disclosure is negative.

Sample size	Fibrils were extracted from human meningeal tissue samples of three different patients.
Data exclusions	No data were excluded from the analyses.
Replication	Cryo-electron microscopy data is based on a single human patient. SDS-PAGE data were replicated successfully in at least three independent experiments.
Randomization	Not relevant to study.
Blinding	Not relevant to study.

Reporting for specific materials, systems and methods

We require information from authors about some types of materials, experimental systems and methods used in many studies. Here, indicate whether each material, system or method listed is relevant to your study. If you are not sure if a list item applies to your research, read the appropriate section before selecting a response.

Materials & experimental systems

n/a	Involved in the study
☐	☒ Antibodies
☒	☐ Eukaryotic cell lines
☒	☐ Palaeontology
☒	☐ Animals and other organisms
☐	☒ Human research participants
☒	☐ Clinical data

Methods

n/a	Involved in the study
☒	☐ ChIP-seq
☒	☐ Flow cytometry
☒	☐ MRI-based neuroimaging

Antibodies

Antibodies used	See Methods: murine 6E10 anti-Aβ antibody (Covance); anti-mouse from goat, horseradish peroxidase conjugated antibody (Dako); in-house rabbit polyclonal antibody directed against Aβ
Validation	*Describe the validation of each primary antibody for the species and application, noting any validation statements on the manufacturer's website, relevant citations, antibody profiles in online databases, or data provided in the manuscript.*

Human research participants

Policy information about studies involving human research participants

Population characteristics	See Methods: Source of human Aβ amyloid fibrils; Age at death; Gender; Diagnosis: AD1: 70 years; female; Alzheimer's Disease (Braak stage VIB) and cerebral amyloid angiopathy/ AD2: 76 years; male; Alzheimer's Disease (Braak stage VI) and cerebral amyloid angiopathy/ AD3: 84 years; female; Alzheimer's Disease (Braak stage VI) and cerebral amyloid angiopathy
Recruitment	Selected based on clinical findings at autopsy.
Ethics oversight	*Identify the organization(s) that approved the study protocol.*

Note that full information on the approval of the study protocol must also be provided in the manuscript.
